# Synergistic Inhibitory Effect of Hyperbaric Oxygen Combined with Sorafenib on Hepatoma Cells

**DOI:** 10.1371/journal.pone.0100814

**Published:** 2014-06-23

**Authors:** Hai-Shan Peng, Ming-Bin Liao, Mei-Yin Zhang, Yin Xie, Li Xu, Yao-Jun Zhang, X. F. Steven Zheng, Hui-Yun Wang, Yi-Fei Chen

**Affiliations:** 1 State Key Laboratory of Oncology in South China, Guangzhou, China; 2 Collaborative Innovation Center for Cancer Medicine, Sun Yat-Sen University Cancer Center, Guangzhou, China; 3 Hyperbaric Oxygen Therapy Center, Affiliated Guangzhou First People's Hospital of Guangzhou Medical University, Guangzhou, China; 4 Department of Hepatobiliary Oncology, Sun Yat-Sen University Cancer Center, Guangzhou, China; 5 Rutgers Cancer Institute of New Jersey, Rutgers University, New Brunswick, New Jersey, United States of America; Texas Tech University Health Sciences Center, United States of America

## Abstract

**Objectives:**

Hypoxia is a common phenomenon in solid tumors, associated with chemotherapy and radiotherapy resistance, recurrence and metastasis. Hyperbaric oxygen (HBO) therapy can increase tissue oxygen pressure and content to prevent the resistance, recurrence and metastasis of cancer. Presently, Sorafenib is a first-line drug, targeted for hepatocellular carcinoma (HCC) but effective in only a small portion of patients and can induce hypoxia. The purpose of this study is to investigate the effect of HBO in combination with sorafenib on hepatoma cells.

**Methods:**

Hepatoma cell lines (BEL-7402 and SK-Hep1) were treated with HBO at 2 atmosphere absolute pressure for 80 min per day or combined with sorafenib or cisplatin. At different time points, cells were tested for cell growth, colony formation, apoptosis, cell cycle and migration. Finally, miRNA from the hepatoma cells was detected by microRNA array and validated by qRT-PCR.

**Results:**

Although HBO, sorafenib or cisplatin alone could inhibit growth of hepatoma cells, HBO combined with sorafenib or cisplatin resulted in much greater synergistic growth inhibition (cell proliferation and colony formation) in hepatoma cells. Similarly, the synergistic effect of HBO and sorafenib on induction of apoptosis was also observed in hepatoma cells. HBO induced G1 arrest in SK-Hep1 not in BEL-7402 cells, but enhanced cell cycle arrest induced by sorafenib in BEL-7402 treated cells. However, HBO had no obvious effect on the migration of hepatoma cells, and microRNA array analysis showed that hepatoma cells with HBO treatment had significantly different microRNA expression profiles from those with blank control.

**Conclusions:**

We show for the first time that HBO combined with sorafenib results in synergistic growth inhibition and apoptosis in hepatoma cells, suggesting a potential application of HBO combined with sorafenib in HCC patients. Additionally, we also show that HBO significantly altered microRNA expression in hepatoma cells.

## Introduction

Hypoxia is a common phenomenon in the solid tumor due to rapid proliferation of cancer cells and/or insufficient blood supply [1]. While cancer cells located in the tumor periphery close to blood vessels can get adequate oxygen to support rapid growth, the cancer cells in the tumor center or inner core are too far away from the vascular system to get sufficient oxygen for survival and often undergo necrosis or apoptosis. Nonetheless, a portion of cancer cells located between the tumor periphery and tumor center, found in a state of low or moderate hypoxia, can survive and enter dormancy by adapting the hypoxic microenvironment. These cells become more malignant and resistant to chemotherapy and radiotherapy because the chemo or radiotherapy mainly kills or inhibits rapidly proliferating cancer cells [2]. The dormant cancer cells play an important role in cancer progression and recurrence because they are the main source for cancer metastasis and/or recurrence. Therefore, stimulating dormant cancer cells by reducing hypoxia in the tumor is a promising strategy for cancer therapy and/or adjuvant therapy.

For many years, hyperbaric oxygen (HBO) therapy, a non-invasion treatment, has been widely used in many common diseases, such as carbon monoxide poisoning, diseases of the nervous system, brain trauma and diabetes mellitus [3]–[5]. HBO can increase oxygen concentration and pressure in the blood and can enhance both the oxygen diffusion rate and effective diffusion distance in tissues, diminishing hypoxia and increasing oxygen levels in the tumor [6]. When the oxygen content is improved, the dormant cancer cells are stimulated resulting in an increased sensitivity to chemo- and radiotherapy. Therefore, HBO can theoretically enhance the effect of chemo- and radiotherapy and reduce recurrence and metastasis by reducing the number of dormant cancer cells. In addition, HBO induces mobilization of stem/progenitor cells from the bone marrow into the peripheral circulation [7], [8], which improves patient recovery. Therefore, HBO not only enhances oxygen content in tissues but also promotes recovery with reduced side-effects and toxicity. In fact, HBO was used for treatment or adjuvant treatment of cancer soon after it was introduced into the clinic in the 1960s [9]. Although there have been debates over HBO therapy for cancer patients in the past few decades [10], [11], many studies show that HBO is an effective therapy for cancer in clinical and experimental models.

Hepatocellular carcinoma (HCC) is the third most common cause of tumor death world-wide, with more than 50% of cases occurring in China [12]. Presently, radical resection is the most common and effective treatment for HCC patients because of poor chemo- and radiotherapeutic response. However, most HCC patients cannot undergo surgical resection because they are already in advanced stages when diagnosed. Radio-frequency ablation, ethanol injection, transarterial chemoembolization and radioembolization are other available treatments currently used for HCC [13]. Regardless of the treatment methods, the overall survival of HCC patients is very poor, at 15%. Thus, there is an urgent need to develop more effective therapies.

Sorafenib, the first oral multikinase inhibitor used for HCC in the clinic recently, inhibits proliferation of cancer cell and blocks angiogenesis [14], [15]. In many preclinical studies and clinical trials, sorafenib was confirmed to effectively inhibit tumor growth while prolonging progression-free survival and overall survival of the cancer patient [16], [17]. Studies indicate that sorafenib inhibits angiogenesis by blocking tyrosine kinase activity of VEGFR2, VEGFR3, PDGFR-β, inhibiting the Raf/MAPK/ERK pathway [18]. Raf/MAPK/ERK pathway regulates cell cycle, gene expression, cell proliferation and differentiation. To date, sorafenib is the first FDA approved drug for the treatment of advanced HCC [19]. We have been treating HCC patients with sorafenib for more than five years and our results indicate that most patients receiving sorafenib have significant survival benefit [20]. However, our clinical data shows less than 10% of HCC patients have complete or partial response to sorafenib and more than half of patients show minor response or stable disease. More importantly, we also observed that drug resistance occurred in many patients after only six months of treatment, which might be produced by sorafenib-induced hypoxia [21]. In addition, most patients have different degrees of adverse or toxic events [22]. Thus, it is most important to increase the efficacy of sorafenib in HCC while reducing side-effects in patients.

In order to increase sorafenib sensitivity in advanced HCC, many researchers explored sorafenib-based combination therapy or sorafenib adjuvant therapy for HCC in vitro, in vivo or clinically [23]–[26]. Although the results showed synergistic effects between sorafenib and certain agents, they were also associated with more severe toxicities. Because HBO can improve oxygen pressure and content in the body and inside the tumor, and mobilizes stem/progenitor cells into the peripheral circulation, it might enhance the effect of sorafenib, preventing drug resistant while reducing adverse or toxic effects. However, sorafenib treatment in combination with HBO has not been reported for HCC. In this study, we treated hepatoma cells with HBO and sorafenib individually or combined. Our results indicate that this combination indeed has beneficial effects, providing experimental evidence for future application in HCC patients.

## Materials and Methods

### Cell Culture

Hepatoma cell lines BEL-7402 and SK-Hep1 purchased from the Institute of Biochemistry and Cell Biology, Chinese Academy of Sciences (Shanghai, China) and ATCC (Manassas, VA), were cultured in RPMI1640 with 10% fetal bovine serum (Invitrogen, USA) at 37°C in a humidified, 5% CO_2_ atmosphere. Cells were passaged at 80%–90% confluency.

### Experimental Design

Hepatoma cells were divided into six groups: the blank control group, cells were treated with the vehicle; HBO group, cells were treated with HBO (see HBO treatment); sorafenib group, cells were treated with 1.6 µM Sorafenib (Selleckchem, USA); cisplatin group, cells were treated with 1 µM cisplatin (Nanjing Pharmaceuticals, China); HBO + sorafenib group, cells were treated with sorafenib followed by HBO one hour later; HBO + cisplatin group, cell were treated with cisplatin and HBO. Cisplatin, a classical chemotherapy agent that inhibits hepatoma cell growth, was used as a positive control. All treatments were started 24 hrs after cells were seeded in 96-well plates.

### HBO Treatment

Cultured hepatoma cells were treated with HBO once daily during the experiment. Cells were placed in a hyperbaric oxygen chamber flushed with a high-flow oxygen (100%) for 20 minutes so that the pressure could reached 2 atmosphere absolute pressure (ATA), and the concentration of oxygen was ≥70%. Then oxygen pressure and concentration were maintained for 40 minutes. Next, the chamber was decompressed until the pressure was reduced to atmospheric pressure. Finally the cells were put into a CO_2_ incubator.

### Cell Proliferation and Colony Formation Assays

Hepatoma cells (0.5∼1×10^3^) in 200 µl were plated into each well of a 96-well plate and incubated overnight. Cell growth in the aforementioned six groups was measured using the cell counting kit-8 (CCK8) assay 4 hours after HBO treatment daily. CCK8 assay was performed according to the manufacturer’s instructions. For colony formation assay, 500 cells were plated into each well of a six-well plate and incubated at 37°C in a humidified, 5% CO_2_ atmosphere for one week followed by staining with 0.05% crystal violet in methanol, in which colonies with size equal to or more than 100 µM were counted.

### Apoptosis and Cell Cycle Assays

Apoptosis assay was performed by fluorescein isothiocyanate-conjugated Annexin V (Annexin V-FICT) and propidium iodide (PI) double staining after cell culture for five days, when a confluence of 80% or more was achieved. The cells were harvested with 0.25% trypsin, washed with PBS and stained with Annexin V-FITC/PI apoptosis kit (Bestbio, China) according to the manufacturer’s instructions and analyzed by flow cytometry (Beckman FC500, USA).

Cells for cell cycle assay were cultured in RPMI serum-free medium for 24 hrs. The medium was changed to RPMI with 10% fetal bovine serum and the cells were cultured for 24 hrs. Cells were collected, fixed in ice-cold 75% ethanol and stored in −20°C for one hour. The fixed cells were washed with PBS, treated using cell cycle kit (Bestbio, China) according to the manufacturer’s instructions and analyzed by flow cytometry (Beckman FC500, USA).

### Transwell of Migration In vitro

1×10^4^ cells in 200 µl of serum-free medium were seeded into the top chamber of a transwell insert (BD Biosciences, USA) and 800 µl of medium containing serum was added into the bottom chamber of a of 24-well transwell plate. The plate was incubated for 14 hrs at 37°C in a humidified 5% CO_2_ atmosphere. Cells migrating from the top to the bottom side of the membrane were stained with 0.05% crystal violet in methanol. Images were acquired using an inverted microscope (Olympus, Japan). Cell counting was carried out by Photoshop software.

### RNA Extraction and Microarray Assay for miRNA Expression

Total RNAs were extracted from hepatoma cell lines with TRIzol reagent kit (Invitrogen, USA) according to the manufacturer’s instructions. The microarray containing 1849 microRNA (miRNA) probes was fabricated in house, hybridized by the labeled total RNA samples, scanned and analyzed by the methods described previously [27].

### Quantitative Reverse Transcription - Polymerase Chain Reaction (qRT-PCR)

To validate the result from the microarray data, reverse transcription (RT) reaction was conducted at 42°C for 60 minutesin a total volume of 25 µL, containing 2 µg total RNA, 5 nM Bulge-Loop specific RT primers (RiboBio Co., Guangzhou, PR China), 0.2 mM each dNTP, 40 U RNase inhibitor, and 200 U M-MLV Reverse Trancriptase (Promega, USA). The RT products were measured by quantitative PCR in a PRISM 7900 HT (Applied Biosystems, USA). This reaction solution contained 2 µL RT products, 2 µL Platinum SYBR Green qPCR SuperMix-UDG reagents (Invitrogen, USA), 500 nM each of Bulge-Loop miRNA forward primer and reverse primer. The quantitative PCR reaction was carried out in a volume of 15 µL as follows: 95° for 2 min, followed by 40 cycles of 95°C for 15 sec and 60° for 30 sec, and with a dissociation temperature. U6 was used as a control for miRNA expression analysis.

### Statistics

Data were represented as a mean with SD. Before multiple groups were compared, normality test and Levene's test would be performed. If data were proven to be normal distribution and homogeneity of variance, it would be analyzed by using one-way ANOVA. If not, Kruskal Wallis test would be used to analyze the data or one-way ANOVA was employed in the analysis after the data was rank-transformed to normality. LSD (least significant difference) *t* test was conducted for post hoc analysis. If two groups were compared, Student *t* tests (2-tailed) would be used.

## Result

### Synergistic Inhibitory Effect of HBO and Sorafenib on the Growth of Hepatoma Cells

**Figure 1:**
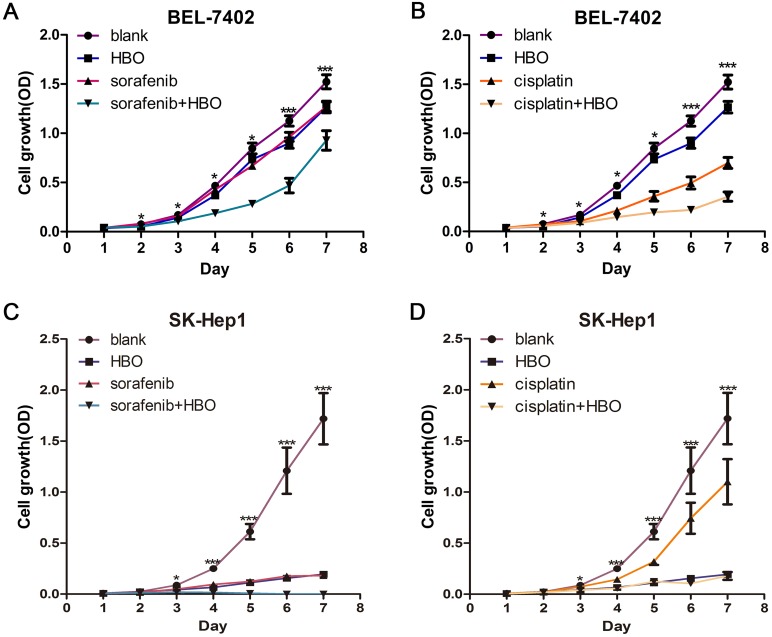
Hepatoma cells were inhibited by HBO, sorafenib, cisplatin and their combinations . (A) BEL-7402 cells were treated with HBO, sorafenib or sorafenib+HBO for one week. Cell proliferation was determined by CCK8 at indicated times (n = 5 for each treatment). The combination of HBO and sorafenib resulted in a marked synergistic growth inhibition in hepatoma cells. Values represent mean ± SD; *P<0.05, ***P<0.001, when compared with the blank group. (B) In BEL-7402 cells, the same synergistic growth inhibition induced by cisplatin and HBO was observed. (C) and (D) In SK-Hep1 cells, synergistic growth inhibition was also induced by the combination of HBO and sorafenib (C) or of HBO and cisplatin (D).

Although Sorafenib is well known to inhibit hepatoma cell growth [28], no study has been reported on HBO inhibition of hepatoma cells. To evaluate whether HBO can inhibit proliferation of hepatoma cells or enhance the inhibitory effect of sorafenib, BEL-7402 and SK-Hep1 hepatoma cells were treated for one week separately with a vehicle (blank control), HBO, sorafenib, cisplatin and combinations as mentioned in Experimental Design. The result showed that HBO, sorafenib or cisplatin individually inhibited the growth of hepatoma cells, as indicated by the lower growth rate in the HBO, sorafenib and cisplatin groups compared to the blank control group, (Fig. 1A–1D). Importantly, HBO plus sorafenib group or HBO plus cisplatin group showed the synergistic inhibitory effect on the growth of hepatoma cells (both p<0.001, Fig. 1A–1D). This result clearly demonstrates that HBO significantly enhances the anticancer effect of sorafenib and cisplatin on hepatoma cells.

### Synergistic Inhibition of HBO and Sorafenib on Colony Formation of Hepatoma Cells

**Figure 2:**
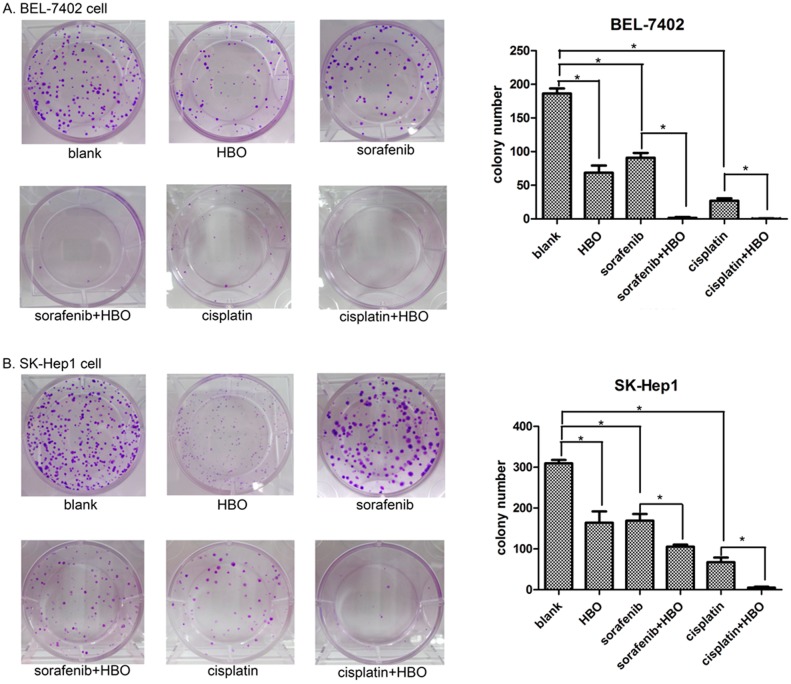
Inhibition of colony formation by HBO, sorafenib, cisplatin or their combinations. (A) The colony formation of BEL-7402 cells was significantly repressed by HBO, sorafenib, cisplatin or their combinations (HBO+sorafenib and HBO+cisplatin, n = 3 for each treatment). The synergistic inhibitory effect of the combinations on hepatoma cells was observed (the values represent mean ± SD, *P<0.05). (B) Colony formation of SK-Hep1 cells was suppressed by HBO, sorafenib, cisplatin or their combinations.

Colony formation is considered a key characteristic of cancer cells [29] and is commonly used in cancer research in vitro. To further evaluate whether the combination of HBO with sorafenib or cisplatin could repress oncogenic growth of hepatoma cell, we performed the colony formation assay. The colonies of hepatoma cells in the HBO, sorafenib or cisplatin treated groups were significantly reduced compared with the blank control group (Fig. 2). When HBO was combined with sorafenib (HBO plus sorafenib group) or cisplatin (HBO plus cisplatin group), the colony number was markedly less than that in the HBO, sorafenib or cisplatin group alone, indicating that HBO significantly enhances the inhibition of colony formation of hepatoma cells by sorafenib and cisplatin (Fig. 2).

### HBO Induces Apoptosis in Hepatoma Cells

**Figure 3:**
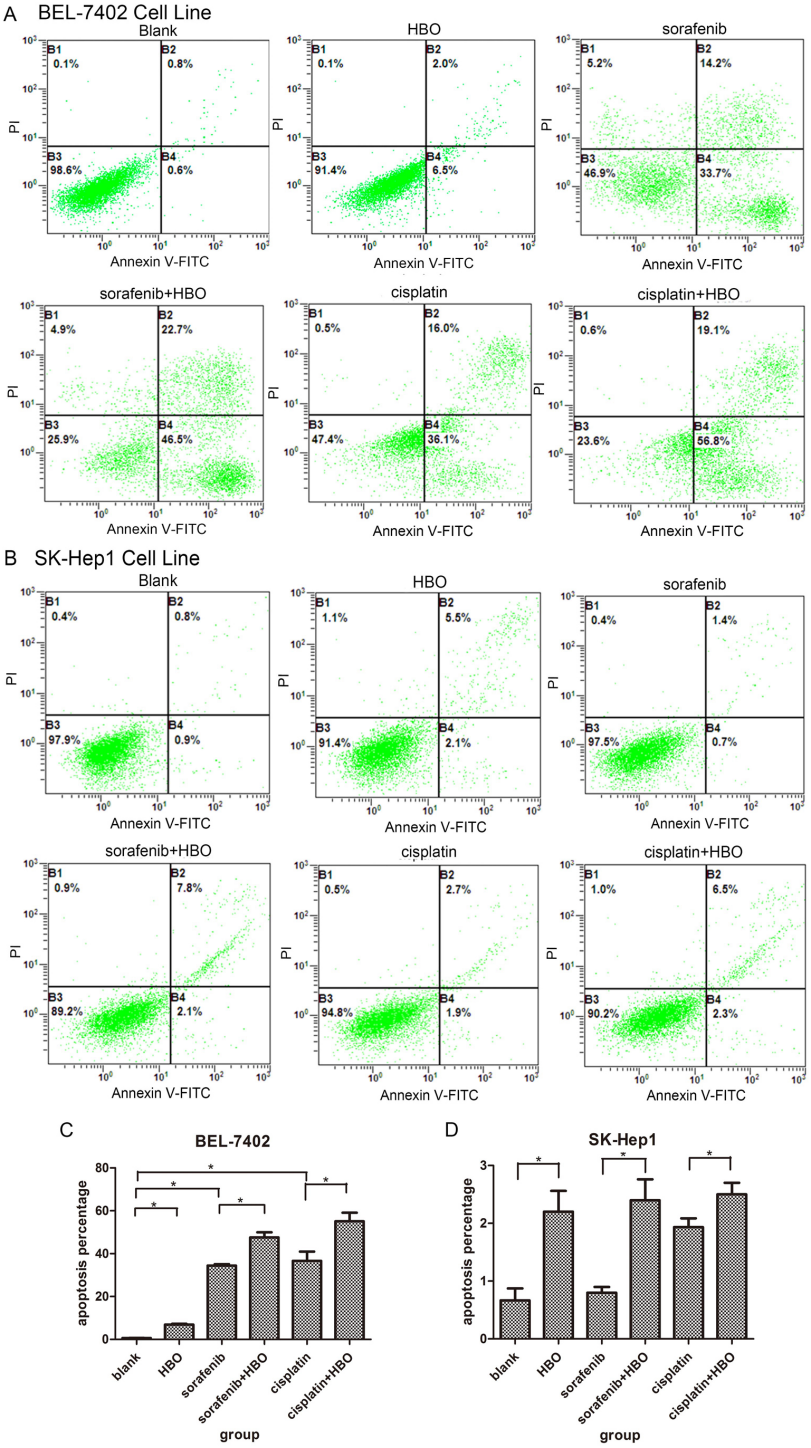
HBO and sorafenib/cisplatin induce apoptosis in hepatoma cells. (A) The representative apoptosis in BEL-7402 cells (n = 3) induced by HBO, sorafenib, cisplatin and combinations was presented and compared with the control. Apoptosis was measured with flow cytometer. Values represent mean ± SD, *P<0.05. (B) The apoptosis in SK-Hep1 cells was analyzed as in figure 3A.

Because HBO, sorafenib and cisplatin repressed growth and oncogenic proliferation of hepatoma cells, we used flow cytometry to analyze their effect on apoptosis and cell cycle progression of hepatoma cells to identify the cause of cell growth inhibition. In BEL-7402 cells, HBO, sorafenib and cisplatin noticeably caused apoptosis compared with the vehicle (Fig. 3A). In contrast to sorafenib or cisplatin, HBO was a weak inducer of apoptosis in BEL-7402 cells. However, HBO strongly enhanced the apoptosis induced by sorafenib or cisplatin in BEL-7402 cells, in which the apoptotic rate in HBO + sorafenib and HBO + cisplatin is much greater than that when treated with HBO, sorafenib or cisplatin individually (Fig. 3A). The synergistic effect of HBO and sorafenib on apoptosis in BEL-7402 is consistent with their synergistic inhibition of the same hepatoma cells. HBO, sorafenib, cisplatin or their combinations only induced minor apoptosis in SK-Hep1 cells (Fig. 3B). Curiously, HBO produced higher apoptosis than sorafenib and cisplatin. The apoptosis rate of SK-Hep1 cells treated with HBO was statistically higher than that of cells treated with vehicle, and HBO could increase the apoptosis induced by sorafenib or cisplatin. This increased apoptosis rate was not significant when compared with HBO alone. These results indicated that the growth inhibition of SK-Hep1 cells induced by these agents does not involve apoptosis.

### HBO Induces Cell Cycle Arrest in Hepatoma Cells

**Figure 4:**
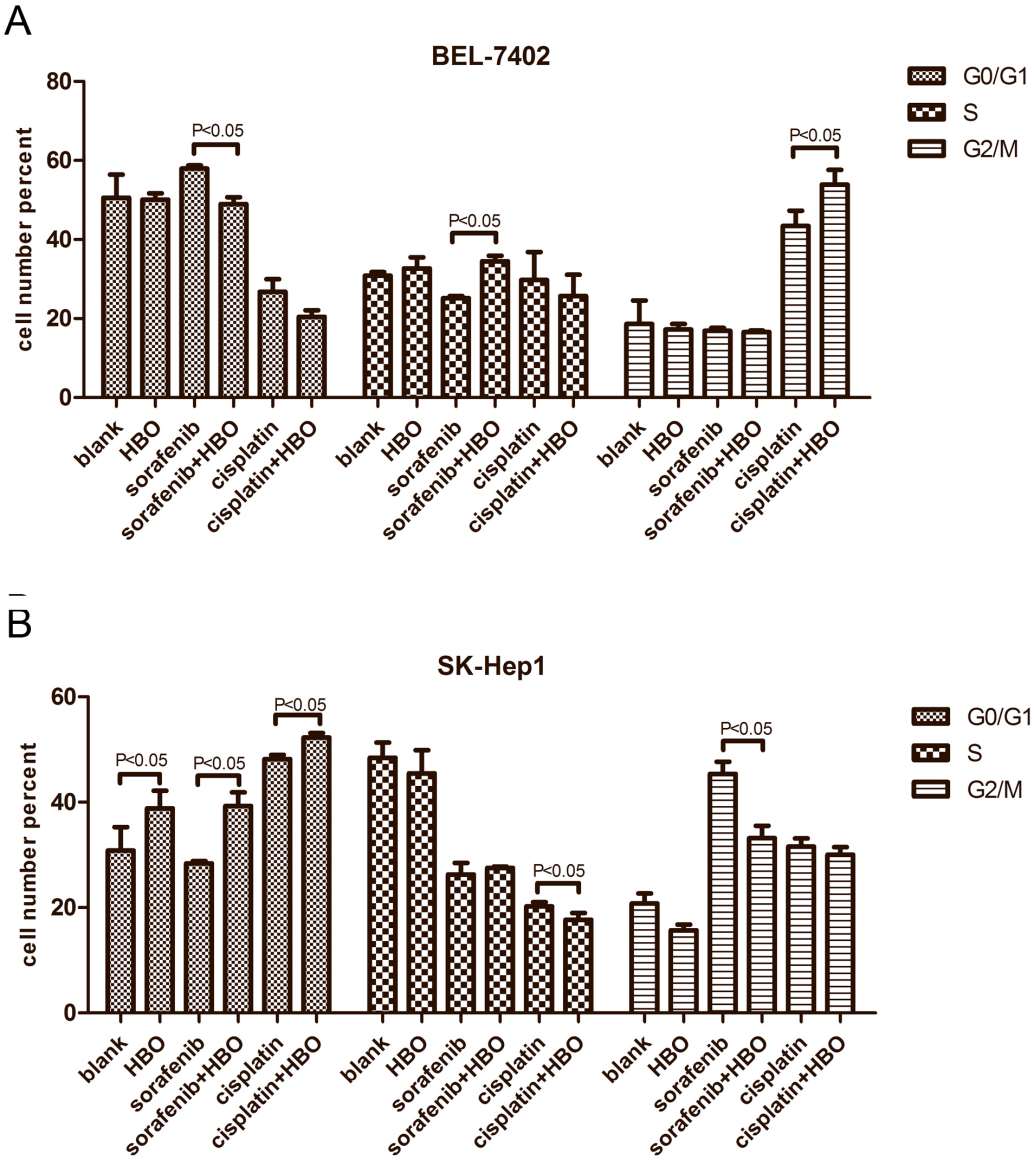
HBO and sorafenib/cisplatin arrest cell cycles in hepatoma cells. (A) The cell cycles arrest of BEL-7402 cells caused by vehicle, HBO, sorafenib, cisplatin and their combinations was presented (n = 3). (B) The cell cycle arrest of SK-Hep1 cells was analyzed as in figure 4A.

We next carried out cell cycle analysis. In BEL-7402 cells, sorafenib caused cell cycle arrest in G1 phase while cisplatin resulted in G2/M arrest (Fig. 4A); although HBO did not alter the cell cycle, it could augment cell cycle arrest induced by cisplatin in BEL-7402 cells (Fig. 4A). However, HBO and cisplatin induced G1 arrest, while sorafenib produced G2/M arrest in SK-Hep1 cells (Fig. 4B). Thus, growth inhibition of SK-Hep1 cells by these agents appears to be mediated by cell cycle arrest not apoptosis. Interestingly, HBO increased the cell cycle arrest by cisplatin but not sorafenib (Fig. 4B), which was consistent with the observed growth inhibitory effect by HBO plus cisplatin on SK-Hep1 cells.

### HBO does not Affect Migration of Hepatoma Cells

**Figure 5:**
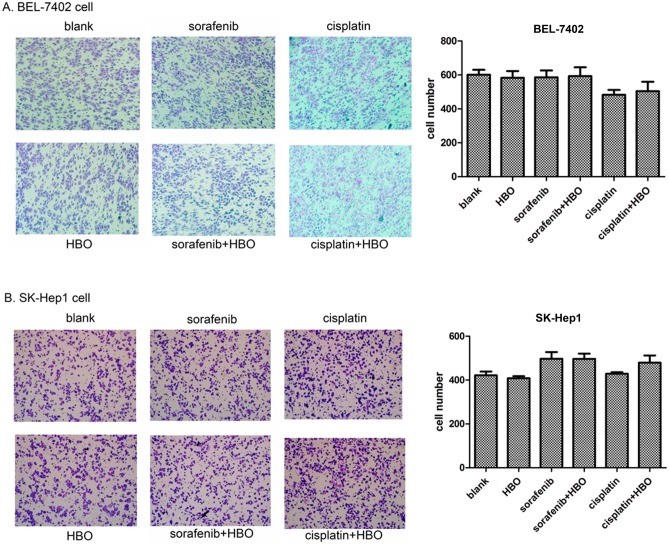
HBO does not affect migration of hepatoma cells. (A) Transwell migration was assayed with BEL-7402 cells at the fifth day after treatment of HBO, sorafenib, cisplatin, sorafenib+HBO or cisplatin+HBO, compared with the blank group. (B) Transwell migration was assayed with of SK-Hep1 cells treated with the same agents and combinations as in figure 5A.

To evaluate effects of these agents on migration of hepatoma cells, transwell migration assays were carried out on BEL-7402 and SK-Hep1 cells. The results showed that the migrated cell number of BEL-7402 or SK-Hep1 cells in the HBO, Sorafenib or Cisplatin group was not significantly different from that in the control group (Fig. 5). Additionally, no difference between Sorafenib and Sorafenib plus HBO or Cisplatin and Cisplatin plus HBO was observed also. These results indicate that HBO, sorafenib and cisplatin do not inhibit migration of hepatoma cells.

### HBO Affects miRNA Expression Profile in Hepatoma Cells

**Figure 6:**
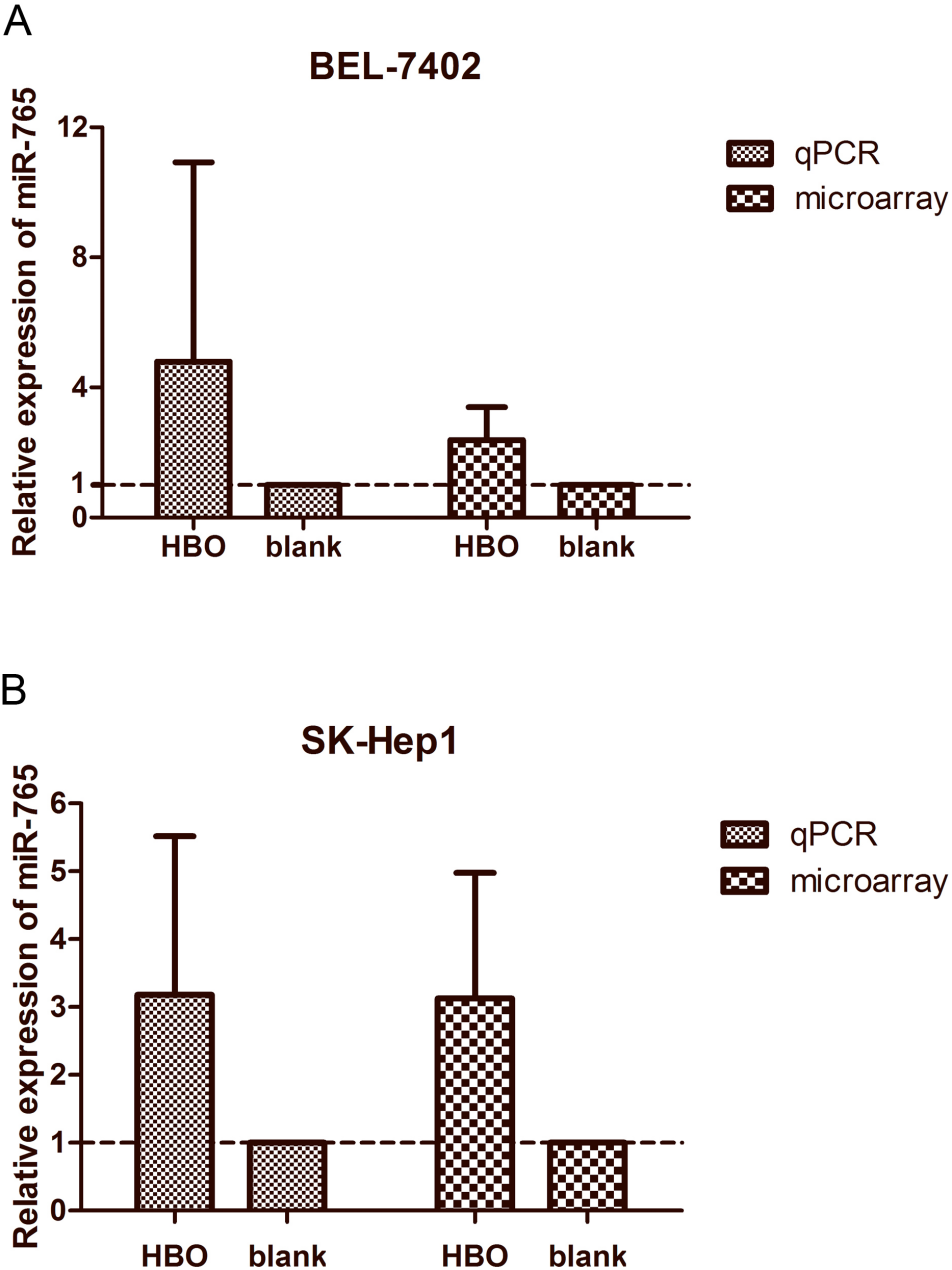
Relative expression level of miR-765 as detected by qRT-PCR and microarray. (A) Relative expression of miR-765 as detected by qRT-PCR in BEL-7402 cells treated with HBO were compared with those assayed by microarray (n = 3). (B) Relative expression of miR-765 in SK-Hep1 cells treated with HBO and the blank (vehicle) as detected by qRT-PCR and microarray (n = 3).

MicroRNA has been reported to be involved in the development and progression of human cancer. Therefore, we asked whether microRNA expression is altered in the HBO-treated hepatoma cells. To this end, the microRNA expression profiles of hepatoma cells from the HBO group and blank control group were determined with microRNA array. The result showed that miR-103a-3p was downregulated, while miR-765 and miR-4428 were upregulated in BEL-7402 and SK-Hep1 cells treated with HBO. The upregulated miR-765 was verified by using RT-qPCR (Fig. 6). These observations suggest that miR-765 and other affected miRNAs play an important role in HBO inhibition of hepatoma cells, an observation worth investigating in the future.

## Discussion

Although it remains unclear whether HBO therapy benefits cancer patients, studies on HBO have shown that HBO treatment for cancer patients is safe. For example, Feldmeier and colleagues reviewed all the published reports from 1960–1993 and found that several studies that reported HBO was enhancing cancer growth, were actually anecdotal. This is in contrast to most studies in the same period that demonstrated HBO treatment did not enhance cancer growth [30]. To date, a large number of studies show that HBO can improve the quality of life for many cancer patients. In 2012, Nakada *et al.,* reported that prostate cancer patients who have radiation cystitis produced by radiotherapy can benefit from HBO treatment, and when HBO treatment was applied for hematuria, earlier treatment was associated with more favorable outcomes [31].

Sorafenib, a multikinase inhibitor that can prolong progression-free survival and overall survival of the cancer patient, has been used in advanced HCC patients for more than 5 years. However, only a small percentage of patients exhibited partial or complete response [32], [33] and the majority of the patients appeared to be drug resistant after receiving six months of treatment. Another limitation for sorafenib is variable side-effects in greater than 80% of patients. Improving curative effectiveness of sorafenib is an important challenge in the clinic currently. In order to find treatment that can enhance efficacy of sorafenib, we tested HBO in combination with sorafenib on hepatoma cells. Cell growth analysis and colony formation assays indicate that HBO not only inhibited hepatoma cells by itself but also significantly augmented the inhibition of sorafenib in hepatoma cells. HBO arrested cell cycle at G1 phase in SK-Hep1 cells but not in BEL-7402 cells, while sorafenib arrested cell cycle at G2/M phase in SK-Hep1 cell and at G1 phase in BEL-7402 cells. In addition, HBO and sorafenib could induce apoptosis. Importantly, HBO and sorafenib synergistically inhibited hepatoma cell growth. These results indicate that growth inhibition of cancer cells by these agents is through different mechanisms. In 2009, Chen *et al.,* reported that treating cells with 100% oxygen at 2.5–3.5 ATA for 6 hr can induce a significant percentage of apoptosis in hematopoietic jurkat and NCI-H929 cells [34]. Kalns JE *et al.,* also found HBO could induce cell cycle arrest at G2/Min in the prostate cancer cell line, LNCaP [35]. These reported results are consistent with our findings and indicate that HBO can induce apoptosis and cell cycle arrest.

The mechanism that HBO potentiates the anticancer effect of sorafenib may be attributed to two aspects. Sorafenib induces apoptosis through inhibition of survival pathways such as STAT3 or Akt [36]–[38] while HBO treatment produces reactive oxygen species, which is also known to mediate apoptosis. Thus, HBO naturally should exacerbate the apoptosis induced by sorafenib. Inhibition of angiogenesis is another potential mechanism. Liu *et al*., found that sorafenib could inhibit the synthesis of hypoxia-inducible factor-1α to exert an antiangiogenic effect in HCC [39]. However, another study indicated that sustained sorafenib therapy led to hypoxia, which could then cause resistance to sorafenib [21]. Thus, reducing hypoxia by HBO may enhance efficiency of sorafenib in cancer patients and reduce sorafenib resistance.

In the early 1960s, HBO was used as a adjuvant therapy to treat a small number of cancer patients [40], but no obvious benefit was observed. Recently, Ohguri *et al.,* reported that non-small-cell lung cancer patients with multiple pulmonary metastases were treated with paclitaxel and carboplatin plus regional hyperthermia and HBO treatments, and the median time for progression of the disease in all patients was eight months, but progression in 16 patients treated with HBO was extended to nine months [41] suggesting there may be benefits from HBO therapy. In an animal experiment, Daruwalla *et al.* found that SMA-pirarubicin when combined with HBO can remarkably reduce liver metastatic tumor nodules of colorectal cancer, and the authors believed that there was a potential benefit of combined therapy using HBO with micellar anthracyclins in colorectal cancer [42]. These studies suggest that HBO may be a safe and effective adjuvant therapy for cancer.

In conclusion, we demonstrate, for the first time, that HBO inhibits the growth of hepatoma cells, induces apoptosis and cell cycle arrest, and strongly augments the inhibitory effect of sorafenib on hepatoma cells. Our results suggest that the combination of HBO with sorafenib may be a promising treatment for HCC patients. Further study is warranted to verify the efficiency of sorafenib combined with HBO using in-vivo preclinical models required for initiation of human clinical trials.
